# Advances in our understanding of anti-PF4 related immunothrombosis

**DOI:** 10.3389/fimmu.2025.1724207

**Published:** 2026-01-21

**Authors:** Luisa Müller, Patrycja Gebicka, Stefan Handtke, Linda Schönborn, Thomas Thiele

**Affiliations:** Institut für Transfusionsmedizin, Universitätsmedizin Greifswald, Greifswald, Germany

**Keywords:** HIT, immunothrombosis, platelet factor 4 (PF4), platelets, VITT

## Abstract

This article focuses on the central role of antibodies against platelet factor 4 (PF4) in mediating immunothrombosis, from classical heparin-induced thrombocytopenia (HIT) to vaccine-induced immune thrombocytopenia and thrombosis (VITT). The latter condition gained international attention during the rollout of vaccines against SARS-CoV-2. Since then, an increased awareness for anti-PF4 mediated disorders arose and patients were recognized with anti-PF4 disorders occurring without prior heparin or adenoviral vector vaccine exposure. These disorders include various acute and chronic VITT-like conditions, i.e. post-viral VITT, diaplacentally transmitted anti-PF4 antibodies in neonatal stroke, monoclonal gammopathies of thrombotic significance (MGTS) and chronic autoimmune VITT of unknown origin. All anti-PF4 related disorders share key serological and immunopathological features with VITT, such as the formation of immune complexes and platelet activation via the Fcγ receptor IIA (FcγRIIA). Via their activation, platelets form procoagulant, aggregatory and secretory phenotypes shaping their interplay with neutrophils, monocytes, and coagulation factors to amplify thrombotic responses. Integrating recent mechanistic insights, clinical observations and diagnostic developments, this review proposes an updated conceptual framework for anti PF4-related immunothrombosis. We aim to raise awareness among clinicians and researchers, to promote early diagnosis and encourage further translational research towards improved therapeutic strategies in this clinically significant area.

## 1 Introduction

Blood coagulation and innate immune responses are crucial to maintain hemostasis and to defend pathogen spreading throughout the circulation. Derailments in these highly regulated processes can lead to immunothrombosis, comprising thrombotic disorders triggered or mediated by immune mechanisms. Within this concept, antibodies directed against platelet factor 4 (PF4)/heparin complexes or PF4 alone gained significant attention to cause the most severe adverse reaction to heparin therapy: heparin-induced thrombocytopenia (HIT) (1); and the adverse reaction to adenoviral vector-based COVID-19 vaccines: vaccine-induced immune thrombocytopenia and thrombosis (VITT) (2–4), respectively.

The research of the pathomechanisms of HIT and VITT raised the awareness and improved the diagnostics of anti-PF4 related immunothrombosis. Meanwhile, “new” forms of anti-PF4 related thrombotic syndromes were discovered, independent of HIT and VITT. Acute VITT-like disorders have entered clinical recognition occuring after virus infections (5–9), and in anti-PF4 related neonatal stroke (10). In addition, chronic anti-PF4 related disorders with recurrent thrombocytopenia and thrombosis were identified. These include chronic monoclonal gammopathy with thrombotic significance (MGTS) (11–13) and chronic recurrent immunothrombosis of unknown origin (14–16). These anti-PF4 antibody-associated disorders were recently reviewed by our group (17).

Here, we provide a contemporary update on the pathomechanisms behind anti-PF4 related immunothrombosis and thrombocytopenia with a focus on downstream activation of platelets and other immune cells. We describe preclinical and experimental data to explain the clinical picture and discuss potential diagnostic and therapeutic implications.

## 2 PF4

In anti-PF4 related immunothrombosis auto-antibodies target PF4. PF4 is a tetrameric protein stored in α-granules of platelets and released upon their activation. Physiologically, PF4 plays a significant role in hemostasis, inflammation and immune regulation by directly activating platelets via the c-Mpl-Jak2 pathway (18). Aside from direct activation, PF4 acts also as pro-inflammatory cytokine exerting chemotactic effects on neutrophils and monocytes (19–21).

The PF4 tetramer has a unique positively charged equatorial band rich in arginine and lysine (22). This is the reason why PF4 can bind with high affinity to negatively charged glycosaminoglycans (GAGs) at endothelial cell surfaces leading to high PF4 concentrations on cell surfaces at sites of injury, promoting coagulation and also to reduce pathogen invasion (23) because PF4 can “tag” various pathogens such as viruses and bacteria (24, 25), probably as part of an early innate immune mechanism. Thereby, the development of anti-PF4 autoantibodies may reflect a misdirected host defense mechanism (24, 26).

Due to their positive charge cloud, two PF4 tetramers usually repel each other. However, polyanions and anti-PF4 antibodies can bind and complex PF4 to form large immune complexes which activate platelets. In HIT, heparin acts as a scaffold that brings multiple PF4 molecules into close proximity, creating new epitopes that are recognized by antibodies (27). In VITT, antibodies bind to the heparin-binding region of PF4 and induce PF4 tetramer clustering independently of heparin, thus forming antigenic complexes on their own (28, 29).

## 3 Anti-PF4 antibodies

### 3.1 Anti-PF4/polyanion antibodies in HIT

HIT is already known for decades (30–32). In HIT, PF4/polyanion complexes induce the production of specific immunoglobulin G (IgG) antibodies (33, 34). Unfractionated heparin is ~10fold more potent to induce HIT compared to low molecular weight heparin (35–37). Also, other negatively charged polyanions like pentosan polysulfate can induce anti-PF4/polyanion antibodies. PF4/polyanion antibodies were described to be polyclonal (29, 38, 39), as the binding of heparin to PF4 induces the formation of multiple neo-epitopes, exposing a complex array of antigenic surfaces (40). However, previous reports indicate that especially pathogenic platelet activating HIT antibodies could be monoclonal (41).

Other sub-entities of HIT exist, where heparin independent anti-PF4 antibodies can be found with coexisting heparin-dependent anti-PF4 antibodies. These antibodies can cause severe prothrombotic clinical conditions, summarized as autoimmune HIT (aHIT) (42, 43). In aHIT, immune complexes are formed by high avidity antibodies leading to conformational changes in PF4 and the presentation of neoepitopes by overcoming charge-related repulsions of PF4 tetramers.

### 3.2 Anti-PF4 antibodies in VITT and VITT-like disorders

Anti-PF4 autoantibodies found in VITT have a high avidity (28). Pathogenic anti-PF4 antibodies are of IgG class, which is why they can be diaplacentally transferred (10). They are also oligoclonal or monoclonal with a remarkable degree of clonal identity of the immunoglobulin light chains (44, 45). Interestingly anti-PF4 antibodies in both, classical VITT after adenoviral vaccination and VITT-like disorders after adenoviral infection, show an extraordinarily high level of similarity underlining a common nature of these immunothrombotic diseases (46). These antibody fingerprints are distinguished by a single immunoglobulin lambda variable 3-21*02 (IGLV3-21*02) light chain paired with a single heavy chain that expresses a shared motif in the heavy-chain third complementarity-determining region 3 (HCDR3) (46). In addition, IGLV3-21*02 light chains show a strongly acidic DDSD motif (consisting of D (aspartic acid) and S (serine)) and a basic K (lysine) to acidic E (glutamic acid) or D mutation at position 31 in light-chain complementarity-determining region 1 (LCDR1) (46).

In VITT and acute VITT-like disorders anti-PF4 antibodies usually decline over time (47). In contrast to acute anti-PF4 related immunothrombosis, chronic VITT-like syndromes were described recently. Due to persistent circulating anti-PF4 antibodies, which differ from the above described antibody fingerprint, patients with chronic VITT-like syndromes clinically present with reoccurring thrombocytopenia and thrombosis. One example is MGTS with a present paraprotein expressing anti-PF4 antibody features (11, 13, 15). Furthermore, chronic autoimmune VITT-like disorders with anti-PF4 antibodies of unknown cause were described previously (16).

Supposedly due to the high avidity of anti-PF4 antibodies in VITT and VITT-like disorders the clinical manifestation is often more pronounced with more thrombotic complication observed than in HIT. In contrast to HIT, these VITT patients often need treatment approaches beyond anticoagulation, as described in section 8. on therapeutic implications beyond anticoagulation.

### 3.3 Laboratory detection and distinction of various anti-PF4 antibodies

Although both HIT and VITT antibodies usually test positive by standard microtiterplate-based anti-PF4/heparin enzyme-immunoassays (EIAs), VITT antibodies are not recognized by common rapid assays for HIT antibodies (48). Gold standard to detect and differentiate between anti-PF4 antibodies are functional assays like the serotonin release assay (SRA) or the heparin-induced platelet activation assay (HIPA), that can be modified by addition of PF4. The latter modification increases the sensitivity to detect platelet activating antibodies directed against PF4 alone. However, these tests are limited to specialized diagnostic laboratories. Currently, two novel tests are reported to be able to differentiate between HIT and VITT antibodies, a fluid-phase EIA (7) and a modified chemiluminescence assay (49). The clinical utility of these approaches may be important given the fact that HIT and VITT-like disorders require different first line treatments. Quantitative assays further enable the monitoring of anti-PF4 levels over time because they can persist over years in chronic VITT (50–52).

## 4 Platelet activation

In all anti-PF4-related immunothromboses, antibodies form immune complexes with their respective antigens (PF4 alone or PF4/polyanion complexes). These immune complexes are the main drivers of the activation of platelets and other immune cells.

Immune complexes activate platelets via the Fcγ receptor IIA (FcγRIIA; also known as CD32a). FcγRIIA is a low-affinity receptor for the Fc region of IgG, present on platelets, monocytes, neutrophils and macrophages (53). In HIT, IgG antibodies bind to PF4/polyanion-complexes and in VITT, IgG antibodies bind to PF4. This creates large immunocomplexes that are able to induce FcγRIIA-dependent platelet activation after FcγRIIA-crosslinking. FcγRIIA-mediated platelet activation promotes aggregation and thrombus formation (54–56). Cross-linking of FcγRIIA leads to changes in the intracellular immunoreceptor tyrosine-based activation motifs (ITAM) (55, 57). This activates Src family kinases (58) and spleen tyrosine kinase (Syk) (59, 60). Downstream signaling involves activation of bruton tyrosine kinase (Btk) (61, 62) and Phospholipase C gamma 1 (PLCγ) (63). PLCγ generates inositol 1,4,5-trisphosphate (IP3) and diacylglycerol (DAG) leading to moderate mobilization of intracellular stored Ca2^+^ and protein kinase C activation (63).

**Figure 1:**
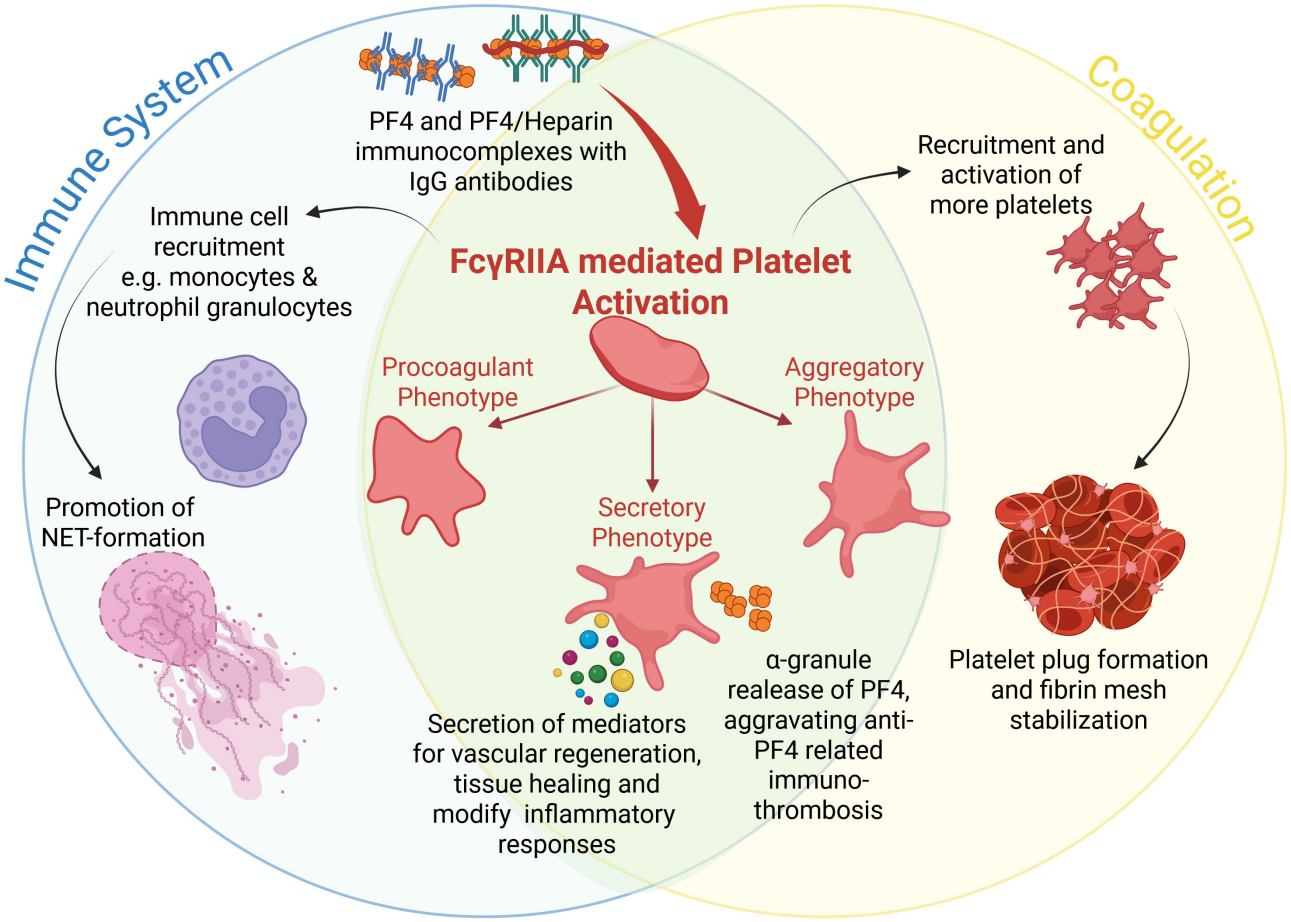
Overview of FcγRIIA mediated platelet activation in anti-PF4 related immunothrombosis. Created in BioRender. Wesche, J. (2025) https://BioRender.com/kh5uw54.

Following platelet activation distinct functional subpopulations of platelets can be observed (64), which can be grouped into aggregatory platelets, procoagulant platelets and secretory platelets. These distinct phenotypes may also play important roles in PF4-mediated immunothrombosis (**Table 1**; **Figure 1**).

**Table 1:** Summary of prominent features in the three platelet phenotypes observed in anti-PF4 immunothrombosis.

Platelet population	Procoagulant	Aggregatory	Secretory
Role in Coagulation	Thrombin boost; Coagulation amplification Thrombus stabilization	Platelet plug formation; Haemostasis	Mediator release (PF4, cytokines) Thrombus stabilization
Trigger	Collagen, Thrombin, Immune compexes	Collagen, vWF, ADP, Thrombin	ADP, Thrombin, Immune complexes
Predominantly described mechanism of activation	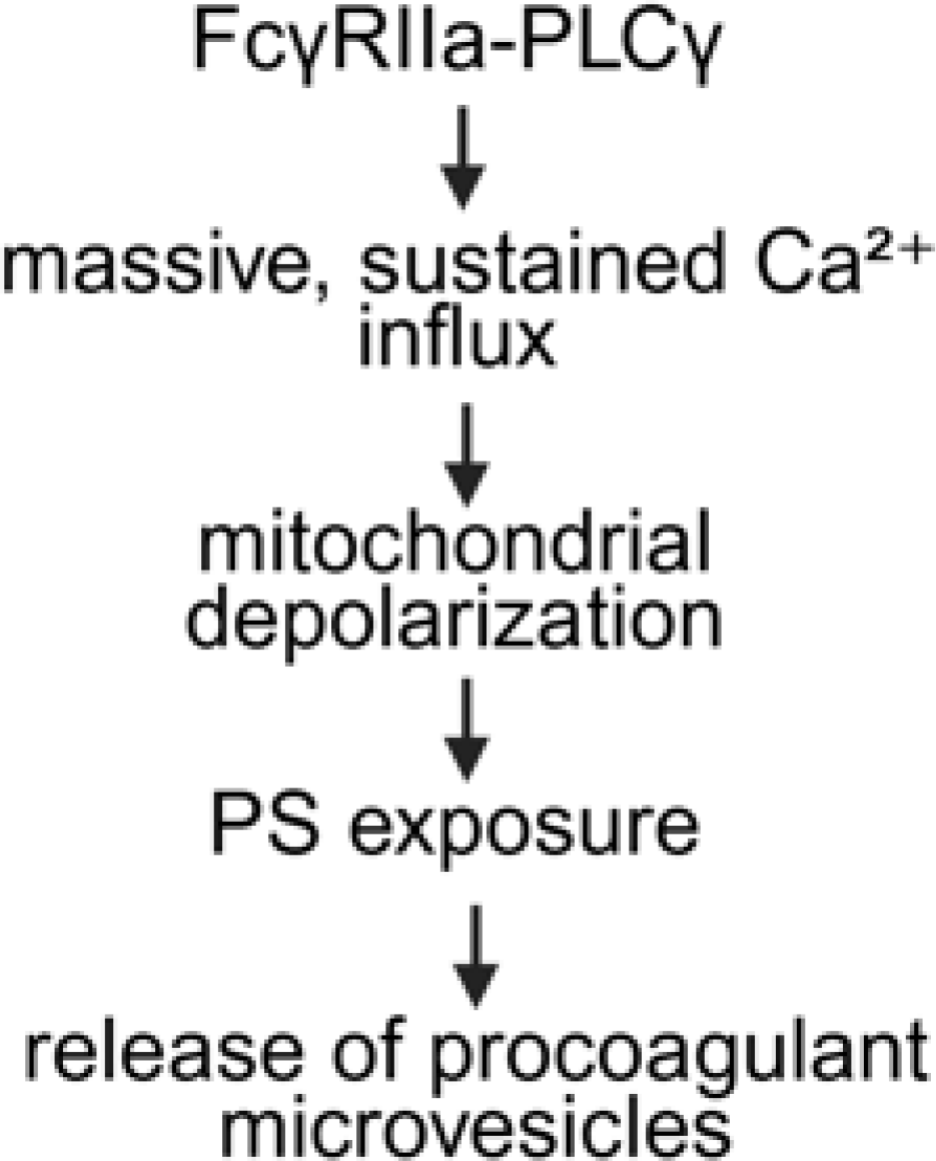	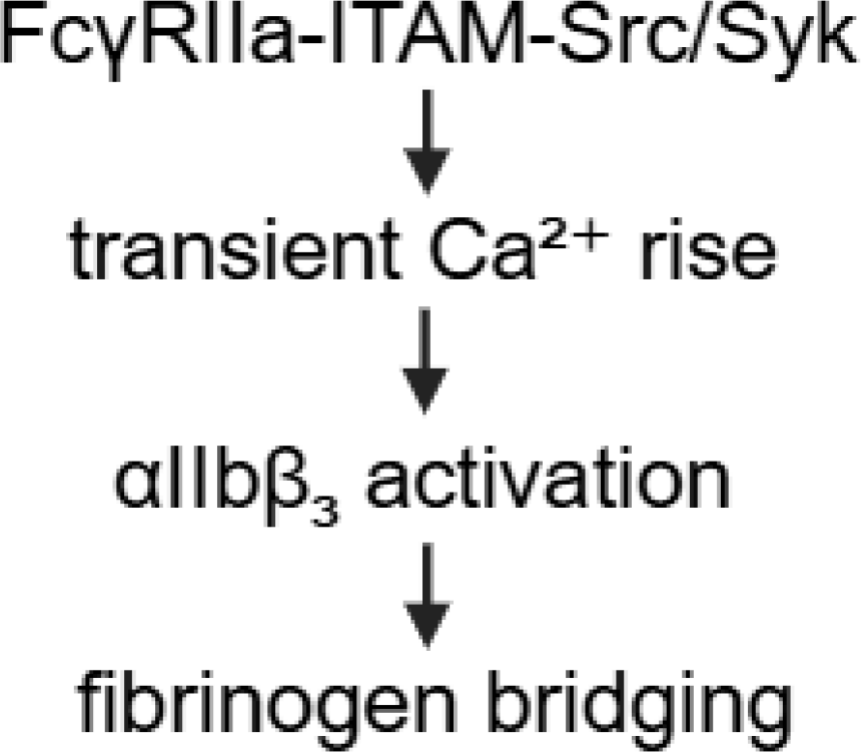	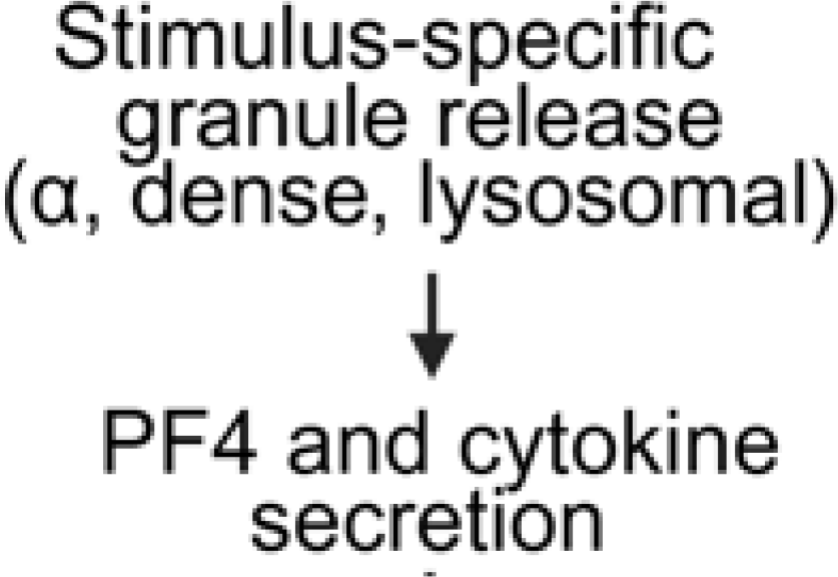
Intracellular Calcium levels	Highly and persistently elevated	Elevated	Stimulus-specific moderate elevation
Morphologic characteristics	Cell swelling (often balloon shaped) with less prominent pseudopods 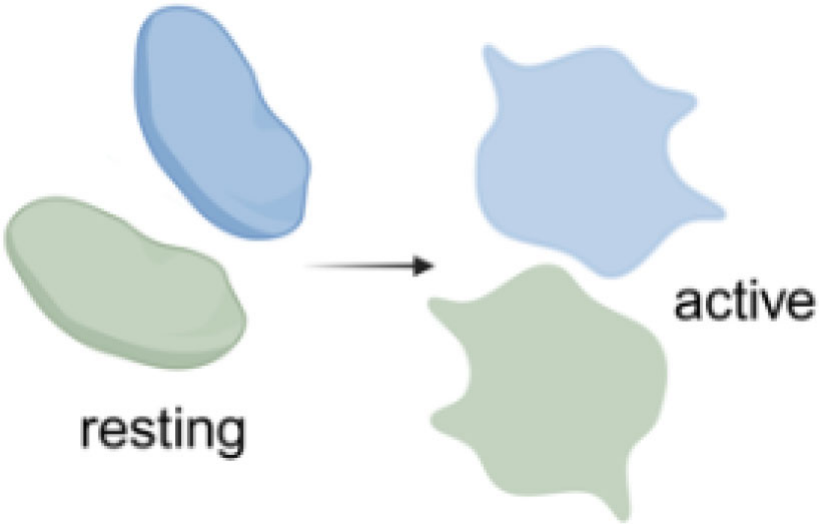	Abundant pseudopods to attract other platelets 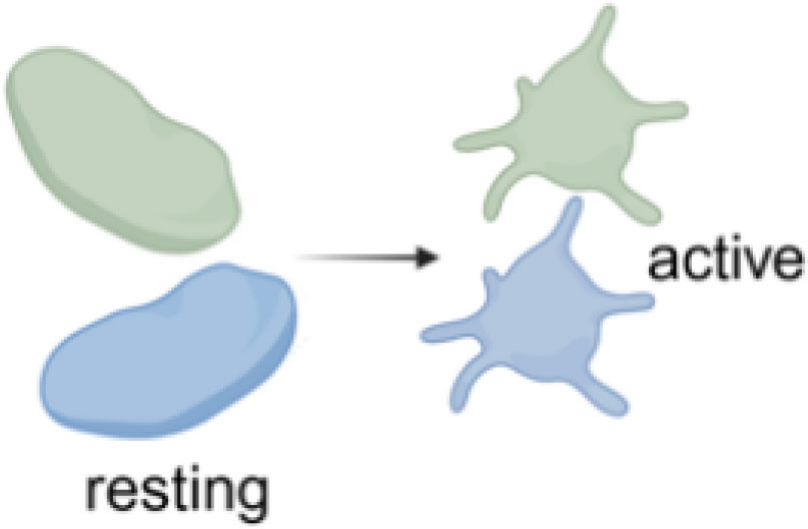	Granule secretion; limited shape change 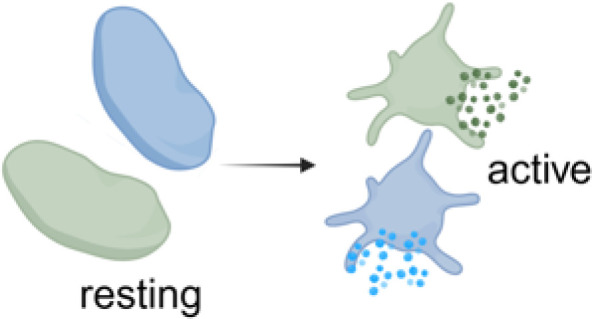
Activation of integrin αIIbβ3	Mostly inactive	Active	Variable, depending on stimulus
Microparticle release	High	Low	Secretory vesicles + mediator release
Suggested role in anti-PF4 related immunothrombosis	- amplification of thrombin generation - promotion of clot formation	- promotion of platelet aggregation and thrombus growth	- release of PF4, which amplifies formation of PF4-anti-PF4 antibody immunocomplexes - sustenance of thrombotic and pro-inflammatory signal cascades

### 4.1 Procoagulant platelets

Procoagulant platelets, sometimes also referred to as collagen-and-thrombin (Coat)-activated/Coated platelets (65–67) are characterized by the externalization of negatively charged phospholipids such as phosphatidylserine (PS) and high levels of P-selectin (CD62P) on their surface (68). Notably, procoagulant platelets typically have inactivated integrin αIIbβ3 (69, 70), rendering them less effective for aggregation. Procoagulant platelets bind coagulation factors and enhance coagulation on their membrane surface (71–75). Exposed PS provides a catalytic platform for the assembly of coagulation factor complexes and thrombin generation (76).

Intracellular Ca^2+^ levels are strongly increased in procoagulant platelets and remain persistently high (77, 78), while lower and less sustained cytosolic Ca^2+^ concentrations generate non-procoagulant platelets (79). Increased cytosolic Ca^2+^ levels are a prerequisite for the development of the typical balloon shape due to cell swelling in procoagulant platelets (80) for surface maximization to drastically increase surface presentation of coagulation factors (80, 81).

Noticeable, FcγRIIA-mediated activation by anti-PF4 immune complexes can drive platelets into a strongly procoagulant phenotype, even without the classical activation pathways as induced by ADP, thrombin, or collagen signaling. Furthermore, the procoagulant platelet phenotype predominantly drives platelet-leukocyte interactions and subsequent thrombus formation in anti-PF4 immunothrombosis (75). In line with these findings, Kaiser et al. (82) showed in murine models and patient samples that procoagulant platelets play a key role in venous thrombosis. They observed that platelets exposing PS are enriched within venous clots, where they support thrombin generation and promote interactions with leukocytes. This provides a mechanistic link to the clinical observation that venous thrombi are common in HIT and VITT, where anti-PF4/heparin antibodies and/or anti-PF4 antibodies drive the formation of procoagulant platelets. Warkentin & Sheppard demonstrated that pathogenic HIT IgG antibodies are able to trigger a procoagulant response in platelets (83), and newer reports on VITT from Althaus et al. similarly describe that anti-PF4 immune complexes increase the generation of procoagulant platelets (84).

### 4.2 Aggregatory platelets

Aggregatory platelets are defined as platelets that primarily contribute to thrombus growth by binding to each other through fibrinogen bridges via integrin αIIbβ3. In contrast to procoagulant platelets, they do not undergo membrane ballooning or PS exposure and display only moderate increases in intracellular Ca²^+^ levels (85, 86). In aggregatory platelets intracellular Ca^2+^ levels are also increased but hundred times less compared to procoagulant platelets (87). The P2X1 receptor mediates the Ca^2+^-increase to facilitate aggregation (88). A proportion of Ca^2+^ is transported into platelet mitochondria to enhance energy production by stimulating a variety of enzymes to fuel the energy-consuming process of aggregation (89).

Morphologically, aggregatory platelets present activated integrin αIIbβ3 (fibrinogen receptor) on their cell surface and stretch out pseudopods to further attract platelets to the site of injury (85, 90–92). In the aggregatory activation pathway, the highly abundant αIIbβ3 integrin plays a major role (93). In resting platelets, αIIbβ3 integrins are inactive. During platelet activation αIIbβ3 becomes activated to support binding to fibrinogen (94), which is central to platelet aggregation (95, 96).

While FcγRIIA is the central receptor mediating immune complex–induced platelet activation, current evidence and reports in literature mainly links this signaling to the formation of procoagulant platelets. A direct contribution of FcγRIIA to the aggregatory platelet phenotype has not yet been described. However, we hypothesize a contribution also of aggregatory platelets to anti-PF4-related thrombosis (**Table 1**, **Figure 1**) because activated αIIbβ3 integrins on aggregatory platelets and their released thrombin enhance thrombus formation, stabilization and growth (97), which could add to the high thrombogenicity of VITT-like syndromes.

### 4.3 Secretory platelets

Another operational mode of platelets is transition to a secretory phenotype (98). Secretory platelets show a targeted release of stored intracellular contents and integrate less strongly within the clots (99, 100). The secretory function centers on three distinct storage compartments: α-, dense granules and lysosomes. α-granules contain fibrinogen, von Willebrand factor, chemokines and growth factors. Notably, PF4 is one of the most abundant proteins in α-granules.

Activation of glycoprotein VI (GPVI) by collagen can induce substantial release of PF4 (98). This secretory response is relevant as GPVI and FcγRIIA work together to amplify platelet activation signals in the presence of immune complexes. Platelets activated by anti-PF4 immune complexes thus release high amounts of the relevant antigen targeted by the corresponding antibodies, which enhances immunothrombosis.

Dense granules contain adenine nucleotides (ADP, ATP), serotonin, and Ca^2+^ ions, while lysosomes provide hydrolytic enzymes to complete the secretory arsenal (101, 102). These mediators not only amplify platelet aggregation and thrombin generation but also modulate vascular permeability and leukocyte recruitment. In this way, the granular release contributes to the thromboinflammatory environment underlying immunothrombosis.

## 5 Plasma proteins

In addition to platelet-derived factors, plasma proteins can interact with PF4-containing immunocomplexes. Krauel et al. showed that fibronectin changes the binding of PF4 and heparin, affecting both the size and the antigenicity of the immune complexes (103). By interfering with the clustering of PF4 and heparin, fibronectin makes it less likely that antibody-binding sites are exposed. This observation supports the idea that plasma proteins may act as natural “buffers” of PF4-complex antigenicity, making them less capable of triggering an immune response. Notably, fibronectin levels correlate inversely with HIT risk: patients with lower plasma fibronectin levels show greater susceptibility to both PF4/heparin immunization and clinical breakthrough of HIT, suggesting that fibronectin levels may serve as a modifiable risk factor in susceptible individuals (103).

Other plasma and extracellular polyanions may also influence PF4 immunogenicity. One example is von Willebrand factor (vWF), which is released from activated endothelium and can bind PF4 along its elongated multimers (104). However, direct *in vivo* evidence for PF4-vWF mediated clot formation in either HIT or VITT is still lacking, so this concept remains hypothetical.

Consequently, the overall composition of plasma proteins may further determine whether PF4 can become a target for pathogenic antibodies in HIT and VITT. The identification of plasma proteins as modulators of PF4 complex antigenicity opens promising directions for targeted modulation of immune complex formation as adjunctive or preventive therapies in anti-PF4 syndromes, complementing standard anticoagulation strategies.

## 6 Immunothrombosis

Immunothrombosis is a conserved physiological defense mechanism of the innate immune system involving the formation of localized blood clots to prevent the spread of microbes and promote tissue repair (105). This process requires the coordinated action of neutrophils, monocytes, platelets, endothelial cells, coagulation factors and regulatory pathways to generate a rapid barrier against pathogens and danger signals (106).

The initiation of immunothrombosis encompasses the activation of pattern recognition receptors, such as Toll-like receptors (TLRs) on immune cells, endothelial cells and platelets. These receptors recognize pathogen-associated molecular patterns and damage-associated molecular patterns (107). Downstream, these signals converge to activate inflammasomes (e.g. NLRP3) in monocytes, macrophages, and endothelial cells, triggering the release of IL-1β and IL-18, and the shedding of procoagulant microvesicles that are rich in tissue factor (TF) and PS (108), creating a procoagulatory environment.

Under physiological conditions, these events help to maintain vascular integrity and control infection without significantly disrupting the blood flow (105). However, excessive or misdirected activation leads to immunothrombosis with tissue injury and organ failure (107). Immunothrombosis occurs usually in sepsis and other systemic inflammatory conditions such as severe viral infections or autoimmune diseases but was also described as a mechanism in anti-PF4 related disorders (17, 24, 109).

Anti-PF4 related immunothrombosis comprise most of the described features but is triggered by platelet-activating immune complexes linking innate immunity and autoimmunity with cellular and plasmatic coagulation (110). Immunothrombosis in anti-PF4 related disorders can occur in both, arterial and venous vessels but is more common in venous vascular beds. In HIT with anti-PF4/heparin antibodies thrombosis is observed in approximately 30-50% of patients (110–114) with deep vein thrombosis and pulmonary embolism representing the typical manifestations and occurring approximately four times more often than arterial events (115). Arterial thrombosis is less frequent but may present as limb ischemia, myocardial infarction, or stroke. The predominance of venous thrombosis likely reflects the combination procoagulant platelet activation and the slower blood flow in veins, which facilitates platelet-leukocyte interactions and fibrin formation (116).

VITT and VITT-like disorders with antibodies against PF4 alone, are characterized by a striking predilection for unusual vascular sites. Patients commonly develop atypical thromboses, such as cerebral venous sinus thrombosis, splanchnic vein thrombosis (including portal and hepatic vein thrombosis), and other unusual venous sites (117, 118). While the precise determinants of this site-specific thrombosis in VITT remain incompletely understood, emerging evidence suggests that enhanced NETosis, altered endothelial permeability, and local tissue-specific factors are relevant to explain this thrombosis pattern.

### 6.1 Neutrophil extracellular traps

One key effector mechanism of immunothrombosis is the release of NETs. These structures consist of decondensed chromatin coated with antimicrobial proteins such as myeloperoxidase, neutrophil elastase, and histones. They were initially described as part of innate immunity, functioning to immobilize and neutralize circulating pathogens. By forming web-like structures, NETs create a physical barrier that traps bacteria, viruses, and fungi, thereby preventing dissemination and promoting pathogen clearance (119).

Beyond their antimicrobial role, NETs exert profound effects on hemostasis. They act as a prothrombotic scaffold by binding fibrin, platelets, and TF, thereby accelerating thrombin generation and fibrin deposition (120, 121).

Histones exposed on NETs are strongly cationic and exert direct procoagulant and cytotoxic effects: they can induce platelet aggregation via TLR 2 and 4 (122), damage endothelial cells to expose more subendothelial matrix which promotes platelet adhesion. NETs also bind and activate factor XII, thus linking neutrophil activation to the intrinsic pathway of coagulation (119). Endothelial injury induced by histones and reactive oxygen species (ROS) released during NETosis enhances vascular permeability and promotes additional leukocyte and platelet recruitment (123) and activation (119–121).

NET formation is potently triggered by anti-PF4 antibodies and PF4 (or PF4/heparin) immune complexes because they directly activate platelets and neutrophils in HIT and VITT (124, 125).

In HIT, NETosis is now recognized as a key driver of thrombosis. Using human HIT immune complexes, Perdomo et al. showed that anti-PF4/heparin antibodies induce NET formation via FcγRIIa on neutrophils and through close interactions between neutrophils and activated platelets (126). Thrombi formed in microfluidic systems and in a murine model contained abundant neutrophils, extracellular DNA, citrullinated histone H3 and platelets. As a proof of principle neutrophil depletion or inhibition of NETosis almost completely abolished clot formation, demonstrating the central role of NETosis (126). Gollomp et al. extended these findings by demonstrating neutrophil accumulation, extensive NET release and a central role of peptidylarginine deiminase 4 (PAD4) in venous thrombosis in a passive immunization model of HIT (124). PAD4 is expressed in granulocytes and is essential for NET formation via PAD4-mediated histone citrullination (127). Additional evidence from *in vivo* and *in vitro* studies showed that HIT associated NETosis depends on production of reactive oxygen species (ROS) and NADPH oxidase 2 (NOX2) (128).

In a landmark study on VITT, Leung et al. (125) demonstrated that anti-PF4 antibodies induce robust NETosis both *in vitro* and *in vivo*. Similar to HIT, NET-formation is driven by PAD4 (125). Using flow cytometry and immunofluorescence, the authors detected elevated NET markers (citrullinated histone H3, extracellular DNA, and myeloperoxidase) in VITT patients’ sera and showed that VITT IgG directly stimulates purified neutrophils to undergo NETosis in the presence of PF4. Furthermore, in a flow microfluidics system mimicking vascular conditions, VITT antibodies induced thrombus formation containing abundant platelets, neutrophils, fibrin, extracellular DNA and citrullinated histone H3, directly recapitulating the histopathological features observed in VITT patient thrombi (125).

Based on these insights, anti-PF4 immune complexes likely trigger a self-sustaining prothrombotic loop: activate platelets and neutrophils, inducing NET release that provides a scaffold for thrombi, damages vessel walls, exposes additional adhesion molecules, attracts more leukocytes and platelets, and amplifies coagulation. Thereby creating a vicious cycle of escalating thromboinflammation characteristic of HIT and especially VITT. Recent insights indicate that crosstalk of NETosis and coagulation can be directly shaped by complement. C1q has been shown to trigger NET formation in primed neutrophils *in vitro*, and these NETs activate coagulation factors FXII and FXI, linking complement to the intrinsic pathway of coagulation (129).

### 6.2 Complement activation

Complement activation represents an important mechanism in anti-PF4 related immunothrombosis. The bidirectional interface between the complement and coagulation cascades is well-established (130, 131). Complement activation can be initiated by coagulation enzymes, and conversely, complement fragments can strongly modulate hemostatic responses. For example, factor XIIa can trigger the classical pathway by activating the C1 complex, while thrombin is capable of directly cleaving C3 and C5 into their active fragments, thereby reinforcing inflammatory and procoagulant amplification loops.

The classical pathway is the predominant route of complement activation by anti-PF4/heparin immune complexes in HIT. *In vitro* studies have demonstrated that HIT immune complexes directly engage C1q, thereby initiating classical pathway activation and driving downstream generation of C3 and C5 activation products (132). This process results in robust deposition of activated C3 fragments on neutrophils and monocytes. Inhibition of proximal classical pathway components such as C1q or C1r or blockade of C3 activation substantially attenuates complement-dependent monocyte tissue factor expression and reduces platelet adhesion to injured endothelium, underscoring the functional relevance of complement in HIT-associated immunothrombosis. Clinical data further support this mechanism. In a recent cohort of HIT patients, complement activation measured by C3 activation products was markedly higher in HIT than in individuals with non-pathogenic anti-PF4/heparin antibodies (133). Importantly, selective inhibition of the alternative pathway, had no measurable effect on C3 or C5 generation by HIT immune complexes, indicating that complement activation in HIT is predominantly classical pathway driven (134).

Evidence for complement involvement in VITT is limited to case studies and mechanistic observations rather than large clinical or broader mechanistic studies. In a detailed report of a single patient with severe VITT, Cugno et al. described profound complement consumption, with markedly elevated levels of terminal complement complex sC5b-9, and biochemical evidence for activation of classical and lectin pathways (135). Further, a small number of VITT patients was treated with the anti-C5 monoclonal antibody eculizumab resulting in clinical improvement (136). However, at this point no firm conclusions can be drawn by these studies (137).

## 7 Diagnostic implications

Improved diagnosis of anti-PF4 related immunothrombosis is crucial for timely and effective patient treatment that massively affects patient outcome. In the majority of patients, platelet-activating anti-PF4-antibodies are transient and non-recurring (138). However, in some patients, platelet-activating antibodies persist, associated with recurrent thrombocytopenia and sometimes with relapse of thrombosis despite therapeutic-dose anticoagulation (50).

Up to now, no certified test specifically designed to detect VITT antibodies is commercially available. Compared to HIT antibodies, VITT antibodies are hardly recognized by rapid HIT assays (48, 49). Even functional tests like the serotonin release assay (SRA, commonly used in North America) and the heparin-induced platelet activation assay (HIPA, commonly used in Europe) show negative or only weakly positive results (27, 139). To improve diagnosis of VITT and newly recognized VITT-like disorders and to better distinguish between anti-PF4 and anti-PF4/heparin antibodies a novel rapid chemiluminescence assay was developed (49). Diagnosis of VITT antibodies can be confirmed by adapted functional assays, the PF4-enhanced SRA (139, 140) or the PF4-induced platelet activation assay (PIPA) (141).

These functional assays are of special importance, as not all detectable antibodies against PF4 are pathogenic and immunoassays display highly varying sensitivity. Based on the above described relevance of the formation of a procoagulant platelet phenotype, novel functional tests center around the analysis of procoagulant platelets in anti-PF4 related immunothrombosis. These functional tests focus on flow-cytometry-based approaches using patient serum/plasma samples in combination with whole blood or isolated platelets of healthy donors. In these tests, procoagulant platelets are identified by surface expression of CD62P in combination with PS (detected by binding of Annexin V) (84, 142, 143). Additionally, GSAO, a cell death marker, has been reported to discriminate activated from procoagulant platelets in combination with CD62P measurement (144, 145). However, it should be noted that GSAO is not yet commercially available and has so far only been synthesized for research purposes. This restricts its use mainly to experimental studies and limits its potential for broader diagnostic application.

## 8 Therapeutic implications beyond anticoagulation

For HIT, VITT and VITT-like disorders the mainstay of treatment is anticoagulation, as summarized in treatment guidelines (137, 146–148). For acute thrombocytopenia and thrombosis in HIT, VITT and VITT-like disorders vitamin K antagonists like warfarin should not be used as they are suspected to increase the risk of microthrombotic complications (137). However, they may be considered for long-term anticoagulation once platelet counts normalize.

In HIT, discontinuation of heparin to interrupt generation of antigenic PF4/heparin complexes is most important. Additionally, HIT patients, regardless of whether thrombosis is present or not, receive therapeutic anticoagulation with alternative anticoagulants is recommended as the prothrombotic effect of HIT is so strong that prophylactic dose anticoagulation is not sufficient to prevent new thrombosis. The choice of alternative anticoagulants follows individual needs, for example the parenteral direct thrombin inhibitors argatroban and bivalirudin are preferred in intensive care settings due to their short half-life, which enables rapid dose adjustments in cases of high bleeding risk or before urgent surgery (146, 149). Intravenous immunoglobulins (IVIGs) are currently not generally recommended in cases of classic HIT but could be beneficial in aHIT settings (150, 151).

In contrast, VITT and VITT-like disorders often require a multifaceted approach as anticoagulation alone is not sufficient for their treatment. For VITT, IVIG has been successfully applied in first-line therapeutic management (137). Anticoagulation for anti-PF4 disorders is discussed in detail in (152). Non-heparin anticoagulants are preferred in VITT and VITT-like disorders, because of the evidence of cross-reacting anti-PF4/heparin IgG as described in 3.3.

Plasma exchange was successfully used to manage severe acute or refractory VITT cases (153–156). In addition, corticosteroids were applied, in a number of VITT patients (156). However, general recommendations for corticosteroids are lacking in current VITT guidelines.

We here discuss further adjunct treatment options targeting specific mechanisms of immunothrombosis.

### 8.1 Prevention of immunocomplex formation

A suitable option to prevent FcγRIIA-mediated platelet activation in anti-PF4 related immunothrombosis is to inhibit the formation of immunocomplexes in the first place. The anticoagulant danaparoid was used successfully to treat HIT (157, 158) and also VITT (159). Besides inhibiting factor Xa via activation of antithrombin (160), danaparoid directly interferes with the PF4/heparin and PF4- antigens (161, 162) and thus prevents immune complex formation.

### 8.2 Prevention of binding to FcγRIIA and downstream signaling

Non-complexed IgG competes with immune complex binding to FcγRIIA. This is the rational for using high dose intravenous IgG in acute anti-PF4 disorders. The high levels of total IgG after IVIG treatment compete with pathogenic anti-PF4 IgG and reduce FcγRIIA dependent cell activation. IVIG also reduces the half-life of pathogenic autoantibodies in HIT and VITT by competitively blocking the neonate Fc receptor (163), which results in faster degradation of the pathogenic antibodies.

Numerous case studies describe the efficacy of IVIG in patients with HIT (14, 150, 164), but due to the lack of larger, randomized trials it should rather be used in special cases of spontaneous or autoimmune HIT which are refractory to anticoagulation. On the other hand, high-dose IVIG treatment is the main treatment in acute VITT, beside anticoagulation (137, 140, 165). IVIG is also applied in acute prothrombotic crises of patients with MGTS. While anticoagulation alone did not change the fatal course in one patient with MGTS, four other patients who received additional IVIG survived (13). Importantly, IVIG can ameliorate the acute prothrombotic effects of anti-PF4 antibodies, but it does not prevent recurrence of thrombosis and thrombocytopenia in MGTS (13).

Another therapeutic target is downstream signaling following FcγRIIA activation in anti-PF4 related immunothrombosis. Here, Syk plays a major role. Therapeutic potential of Syk-inhibition in HIT was shown *in vitro* by significantly reduced HIT antibody‐mediated platelet activation and monocyte procoagulant activity (166). In a murine model of HIT the Syk-inhibitor PRT-060318 successfully inhibited platelet aggregation (167). First ex vivo data using the Syk inhibitors entospletinib or lanraplenib showed significantly reduced procoagulant platelet and thrombus formation in VITT (168). Furthermore, specific Syk-inhibition in platelets significantly reduced platelet-leukocyte interaction *in vitro* (75).

Btks are promising new targets to reduce FcγRIIA activation (169). Inhibition of Btk by ibrutinib has proven successful in a 37-year-old female with persisting VITT-like antibodies causing arterial and venous thrombotic complications over 13 years (16). Ibrutinib was also applied successfully in a patient with chronic MGTS (13).

### 8.3 Multiple myeloma therapy in MGTS patients

Due to recurrent thrombocytopenia and possible life-threatening thrombotic complications, plasma-cell–directed therapy with daratumumab–bortezomib–dexamethasone is another therapeutic approach for MGTS patients (13, 170). This therapeutic approach should eliminate the corresponding plasma cell producing the pathogenetic anti-PF4 antibodies. Consequently, platelet counts normalized and no subsequent thrombosis occurred in the follow up for one year after treatment (13, 170). However, there is one case described where this approach did not lead to a clinical improvement because the M protein (bearing the anti-PF4 activity) remained and thrombocytopenia persisted (13). In this patient, using ibrutinib to inhibit Btk-mediated platelet activation led to clinical improvement and an increase in platelet count.

### 8.4 Future therapeutic perspectives: targeting immunothrombotic pathways

Many other approaches could interfere more directly with the immunothrombotic mechanisms. Inhibition of complement activation for instance could be such a strategy. The C5 blocker eculizumab has shown clinical benefit in severe or refractory cases of VITT (137). Recent data also indicate that complement activation closely correlates with platelet and neutrophil activation in HIT, supporting its role not only as a biomarker but also as a potential therapeutic target (133).

Another promising approach is the modulation of NET formation or the enhancement of their degradation and clearance. Experimental work in models of immunothrombosis and sepsis suggests that promoting NET degradation by DNase treatment can reduce vascular injury and thrombus formation (119, 128). Although this concept has not yet been systematically investigated in HIT or VITT, NETosis is a promising target to attenuate the inflammatory drive of thrombosis.

Finally, reducing the formation of procoagulant platelets may be feasible. It can be achieved by decreasing supramaximal Ca^2+^ signaling in order to block the formation of mitochondrial ROS (87, 171). This may attenuate platelet hypercoagulability and reduce their prothrombotic action in anti-PF4 related immunothrombosis.

Together, these emerging concepts illustrate how a deeper understanding of the immunothrombotic cascade opens new lanes for the development of adjunctive, mechanism-based therapies of anti-PF4 related immunothrombosis. This will push new concepts for treating these severe diseases and complement conventional anticoagulation therapies.

## 9 Further perspective

The drivers behind immunothrombosis involve complex pathways of immunity and coagulation. Beside anti-PF4 related disorders additional entities of auto-antibody mediated immunothrombosis exist, such as antiphospholipid syndromes (109) and anti-histone antibody (172) associated immunothrombosis. A deeper understanding of the mechanisms will allow to create new diagnostic and therapeutic interventions and further improve our management of anti-PF4 related disorders and other forms of immune thrombosis.
